# Lacunes may worsen cognition but not motor function in Parkinson's disease

**DOI:** 10.1002/brb3.2880

**Published:** 2022-12-31

**Authors:** Keke Chen, Zhaohui Jin, Jinping Fang, Lin Qi, Cui Liu, Ruidan Wang, Yuan Su, Hongjiao Yan, Aixian Liu, Jianing Xi, Boyan Fang

**Affiliations:** ^1^ School of Beijing Rehabilitation Medicine Capital Medical University Beijing China; ^2^ Parkinson Medical Center, Beijing Rehabilitation Hospital Capital Medical University Beijing China

**Keywords:** cognition, lacunes, motor function, Parkinson's disease

## Abstract

**Background:**

As one of the imaging markers of cerebral small vessel disease, lacunes has received little attention. The objective of this study was to investigate the associations of lacunes, cognition and motor function in patients with Parkinson's disease (PD) and whether these associations are independent of other imaging markers.

**Methods:**

Patients were consecutively included from April 2019 to July 2022 in _Beijing Rehabilitation Hospital._ All patients underwent brain magnetic resonance imaging scans, clinical scale evaluations, and neuropsychological tests, as well as quantitative evaluation of postural control. To eliminate the possible factors contributing to cognition and motor dysfunction in patients with PD, in particular white matter hyperintensities and enlarged perivascular space in the basal ganglia, multivariate linear regression models were constructed to sort out the effect of lacunes.

**Results:**

Ninety‐four patients were included in this study, 56 without lacunes and 38 with lacunes. Patients with lacunes showed shorter disease duration, slower gait speed and spent more time on Trail‐Making Test part A (TMT‐A) than those without lacunes. The number of lacunes were positively correlated with the time to complete the TMT‐A and negatively related to gait speed. Multivariate linear regression models showed that the presence of lacunes was associated with longer TMT‐A time after adjusting for potential confounders.

**Conclusions:**

Lacunes were independently associated with worse visual scanning, attention, and processing speed in patients with PD. In addition, lacunes may accelerate the course of PD. Early treatment of vascular disease provides an alternate way to mitigate some motor and cognitive dysfunction in patients with PD.

## Graphical abstract

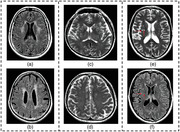

## Highlights

- Lacunes were independently associated with worse visual scanning, attention, and processing speed in patients with Parkinson's disease (PD).
- Lacunes may accelerate the course of PD.
- Early treatment of vascular disease provides an alternate way to mitigate some motor and cognitive dysfunction in patients with PD.

## 1 INTRODUCTION

Parkinson's disease (PD), featured by bradykinesia, rest tremor, rigidity, and gait/posture instability (Postuma et al., 2015), is a neurodegenerative, chronic, and progressive disease. Loss of dopaminergic neurons within the substantia nigra pars compacta and abnormal aggregated α‐synuclein that forms Lewy bodies are the main pathological features of PD (Kalia & Lang, 2015). In addition, vascular pathology may play an important role in its progression by contributing to motor and cognitive dysfunction (Malek et al., 2016; Rektor et al., 2009).

The association between cerebral small vessel disease and PD has received increasing attention in recent years. Cerebral small vessel disease may worsen cognition and motor function in PD (H. Chen et al., 2020; H. M. Chen et al., 2021; Shibata et al., 2019; H. Wan et al., 2022), especially white matter hyperintensities (WMH) (Linortner et al., 2020; Pozorski et al., 2019) and enlarged perivascular space in the basal ganglia (BG‐EPVS) (Park et al., 2019; Shin et al., 2021). Studies have found that moderate‐to‐severe WMH and BG‐EPVS were associated with worse cognition and motor function in PD (Chung et al., 2019; Huang et al., 2020; Park et al., 2019). Lacunes are another imaging markers of cerebral small vessel disease and have been reported to frequently occurred in patients with PD, with a prevalence of approximately 50% (G. Zhang et al., 2016). Evidence has showed that lacunes were associated with mild parkinsonian signs (Camarda et al., 2019; Hatate et al., 2016) and incident parkinsonism (van der Holst et al., 2015). However, the association between lacunes and cognitive and motor dysfunctions in PD has been poorly investigated and results were conflicting. Some studies reported associations with cognitive impairment and motor symptoms in patients with PD (H. Chen et al., 2020; H. M. Chen et al., 2021; G. Zhang et al., 2017; M. Zhang et al., 2022), whereas others did not (Malek et al., 2016; Shibata et al., 2019; Song et al., 2013; Y. Wan et al., 2019). In older adults with vascular diseases, lacunes are strongly associated with WMH and the contribution of lacunes to clinical symptoms will be attenuated by WMH and BG‐EPVS (Ghaznawi et al., 2019; Passiak et al., 2019). However, few studies have considered WMH and BG‐EPVS as possible confounders in analyses investigating the relationship between lacunes and clinical outcomes in PD.

This study aims to exploring the associations of lacunes, cognition and motor function in patients with PD and whether these associations are independent of other imaging markers of cerebral small vessel disease. On the basis of previous literature, we hypothesize that lacunes are independently associated with cognitive impairments in patients with PD.

## 2 METHODS

### 2.1 Subjects

In this cross‐sectional study, we used data from an ongoing cohort named Multidisciplinary Rehabilitation Registration Study on Parkinson's Disease (number: ChiCTR2000033768). Informed consent was obtained either from the patients or their immediate family prior to enrollment.

Inclusion criteria were the followings: (1) diagnosed with clinically established or clinically probable PD according to the Movement Disorder Society clinical diagnostic criteria (Postuma et al., 2015); (2) aged 55 or older; (3) Hoehn–Yahr stage (Hoehn & Yahr, 1967) ≤ 3; (4) had complete 3T magnetic resonance imaging data, including T1‐weighted magnetic resonance images, T2‐weighted magnetic resonance images, and fluid attenuated inversion recovery magnetic resonance images. The exclusion criteria were: (1) diagnosis was uncertain or suspected to be Parkinson's syndrome; (2) history of any other severe brain incident, such as stroke or brain surgery; (3) unable to complete the magnetic resonance imaging examination; (4) osteoarthritis, lumbar disc herniation, fractures, etc. that may affect walking ability; (5) participants with vestibular diseases; and (6) serious visual or hearing impairment.

### 2.2 Clinical assessments

Demographic profiles, medical history, and PD related information were collected. Levodopa‐equivalent doses were computed according to the formula provided by Tomlinson et al. (2010).

Global cognitive function was evaluated using the Montreal Cognitive Assessment (MoCA). Information processing speed was assessed using Symbol Digit Modalities Test (SDMT) (Smith, 1982). The Chinese version of the Trail‐Making Test (TMT) was used to evaluate cognitive elements including visual scanning, attention, processing speed, set‐shifting, and cognitive flexibility (Wei et al., 2018). The severity of motor symptoms and motor complications were evaluated by the Movement Disorder Society United Parkinson's Disease Rating Scale Part III (MDS‐UPDRS III) and Part IV (MDS‐UPDRS IV), respectively. Semiquantitative clinical rating scales including the 10‐Meter Walk Test (10MWT) (Lang et al., 2016) and five times sit‐to‐stand test (FTSTS) (Duncan et al., 2011) were performed to assess patients’ walking ability as well as balance. Four tasks on the C‐Mill (Motek, the Netherlands) were used to evaluate postural control, namely standing with eyes open, standing with eyes closed, tandem stance, and standing on one leg (details in Table A1). The outcome measure was the displacement of the center of pressure (cm/s) during these four tasks. The lower the value, the better the postural control. All assessments were made in the ON state (1−2 h after medication) (Fathipour‐Azar et al., 2022).

**TABLE 1:** Demographic profiles of the study sample

	PD without lacunes (n = 56)	PD with lacunes (n = 38)	p Value
Male, n, %	22/56 (39.3%)	20/38 (52.6%)	.202
Age, years ^b^	63 (8)	64 (7)	.224
BMI, kg/m^2^ ^b^	23.91 (3.75)	25.18 (3.46)	** .002**
Hypertension, n, %	11/56 (19.6%)	12/38 (31.6%)	.186
Diabetes, n, %	3/56 (5.4%)	3/38 (7.9%)	.949
Hyperlipidemia, n, %	7/56 (12.5%)	2/38 (5.3%)	.416
Education years ^b^	12 (6)	12 (5)	.230
Hoehn–Yahr stage			.435
Stage 1.5, n, %	6/56 (10.7%)	1/38 (2.6%)	
Stage 2, n, %	26/56 (46.4%)	18/38 (47.4%)	
Stage 2.5, n, %	9/56 (16.1%)	9/38 (23.7%)	
Stage 3, n, %	15/56 (26.8%)	10/38 (26.3%)	
Disease duration, months ^b^	72 (48)	58 (37)	**.012**
LEDs, mg ^b^	500.0 (350)	431.3 (306.3)	.277
Freezing of gait, n, %	8/56 (14.3%)	5/38 (13.2%)	.876
MDS‐UPDRS IV	2.0 (5.0)	0.0 (3.0)	.145
Fazekas score > 2, n, %	11/56 (19.6%)	13/38 (34.2%)	.112
BG‐EPVS rating ≥ 2, n, %	15/56 (26.8%)	17/38 (44.7)	.071

^a^ Normally distributed data are expressed as the mean ± standard deviation.

^b^ Nonnormal data are reported as the median (interquartile range). Bold values mean p < 0.05.

PD, Parkinson's disease; BMI, body mass index; LEDs, levodopa‐equivalent doses; MDS‐UPDRS IV, Movement Disorder Society United Parkinson's Disease Rating Scale Part IV; BG– EPVS, enlarged perivascular space in the basal ganglia.

### 2.3 Magnetic resonance imaging data acquisition and CSVD evaluation

Magnetic resonance imaging data containing T1‐weighted magnetic resonance images, T2‐weighted magnetic resonance images and fluid attenuated inversion recovery magnetic resonance images were obtained from a GE SIGNA Pioneer 3.0‐Tesla scanner. The parameter settings of each magnetic resonance imaging sequence were as follows:

1. T1‐weighted MR images: repetition time (TR) = 500 ms, echo time (TE) = 10 ms, flip angle = 90°, field of view = 240 × 240 mm^2^, matrix size = 320 × 256 pixels, slice number/thickness = 20/5.0 mm;
2. T2‐weighted MR images: TR = 4500 ms, TE = 100 ms, flip angle = 90°, field of view = 240 × 240 mm^2^, matrix size = 320 × 256 pixels, slice number/thickness = 20/5.0 mm;
3. Fluid attenuated inversion recovery MR images: TR = 15,000 ms, TE = 100 ms, inversion time (TI) = 3000 ms, flip angle = 90°, field of view = 240 × 240 mm^2^, matrix size = 320 × 256 pixels, slice number/thickness = 20/5.0 mm.

**FIGURE 1:**
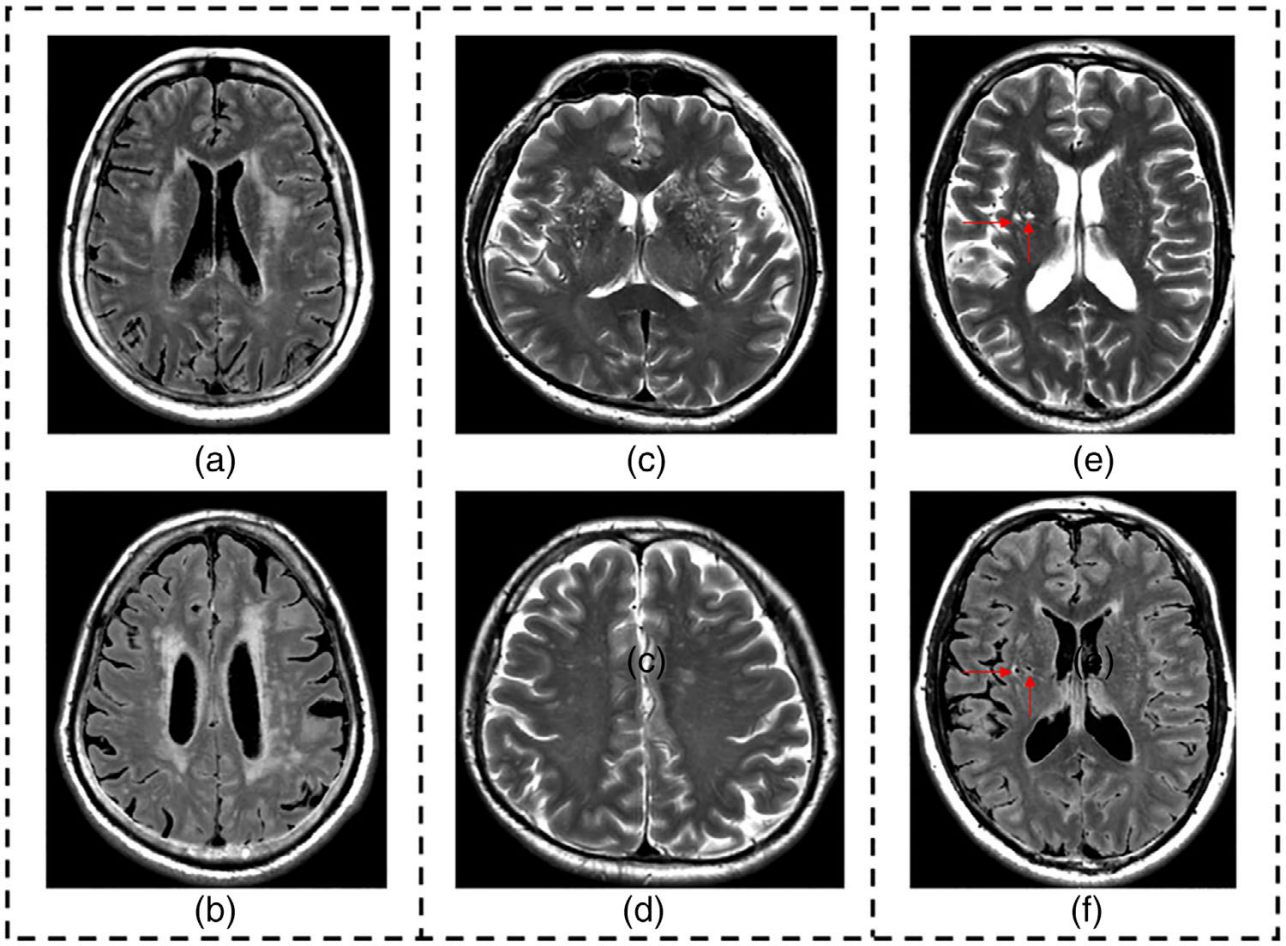
Imaging markers of cerebral small vessel disease. (a) Deep white matter hyperintensities; (b) periventricular white matter hyperintensities; (c) enlarged perivascular space in the basal ganglia; (d) enlarged perivascular space in the centrum semiovale; (e) lacunes on T2‐weighted magnetic resonance images (red arrows); (f) lacunes on fluid‐attenuated inversion recovery images (red arrows)

Lacunes were evaluated by two raters independently according to the STandards for ReportIng Vascular changes on nEuroimaging (STRIVE) (Wardlaw et al., 2013). A lacune was defined as a round or ovoid, subcortical, fluid‐filled (similar signal as cerebrospinal fluid) cavity, of between 3 mm and about 15 mm in diameter (Wardlaw et al., 2013). On T2‐weighted magnetic resonance images, lacunes have a clear cerebrospinal fluid‐like hyperintensity; On fluid‐attenuated inversion recovery images, lacunes generally have a central cerebrospinal fluid‐like hypointensity with a surrounding rim of hyperintensity (Figure 1). The total number of lacunes was recorded. The Fazekas scale was used to rate the severity of WMH (Fazekas et al., 1993). The severity of EPVS in the basal ganglia (BG‐EPVS) was evaluated according to the method proposed by Doubal et al. (2010). WMH was further classified as either no‐to‐mild (Fazekas score 0–2) or moderate‐to‐severe (Fazekas score 3–6) WMH for analysis (Figure 1) (H. Chen et al., 2022). BG‐EPVS rating ≥ 2 was classified as moderate‐to‐severe BG‐EPVS. The interrater agreement was good (the intraclass correlation coefficient was 0.867), and the final consistent results of the two raters was used for analysis.

### 2.4 Statistical analysis

Data normality was verified using the Kolmogorov–Smirnov test (for PD without lacunes group, *n* = 56) and Shapiro‐Wilk test (for PD with lacunes group, *n* = 38). Comparisons of demographic profiles, cognition and motor symptoms between groups were performed using the two‐sample *t*‐test or Mann–Whitney test for continuous variables, the chi‐square test for dichotomous variables, and the Jonckheere–Terpstra test for ordered categorical variables (Hoehn–Yahr stage). Spearman's correlation was used to investigate the relationship between clinical outcomes and the number of lacunes. To evaluate the association between lacunes and PD symptoms, two multivariate linear regression models were conducted. Model 1 was adjusted for potential confounders and those demographic variables that showed significant differences between the two groups, on univariate analysis. Model 2 was further adjusted for WMH and BG‐EPVS. The regression formulas are as follows:

$$\begin{array}{ccc} Model\;1:\;Y\; & = & \;X_{the\;presence\;of\;lacunes\;}+X_{age}+X_{sex}+X_{BMI}+X_{hypertension}\\ & & +X_{disease\;duration}+a \end{array}$$

$$\begin{array}{ccc} Model\;2:\;Y\; & = & \;X_{\mathrm{the}\;\mathrm{presence}\;\mathrm{of}\;\mathrm{lacunes}\;}+X_{\mathrm{age}}+X_{\mathrm{sex}}+X_{\mathrm{BMI}}\\ & & +X_{\mathrm{hypertension}}+X_{\mathrm{disease}\;\mathrm{duration}}+X_{\mathrm{WMH}}+X_{\mathrm{BG}-\mathrm{EPVS}}+a \end{array}$$

*Y* represents cognitive and motor measures (dependent variables) and *a* is a constant term. We constructed 12 linear models of Model 1 and Model 2, respectively. And for each model, the dependent variable (*Y*) was one of the 12 cognitive and motor measures collected. For example, we analyzed:

$$\begin{array}{ccc} \mathrm{Model}\;1\_\mathrm{TMTA}:\mathrm{TMT}-A & = & X_{\mathrm{the}\mathrm{presence}\mathrm{of}\mathrm{lacunes}}+X_{\mathrm{age}}+X_{\mathrm{sex}}+X_{\mathrm{BMI}}\\ & & +X_{\mathrm{hypertension}}+X_{\mathrm{disease}\;\mathrm{duration}}+a \end{array}$$

$$\begin{array}{ccc} \mathrm{Model}\;2\_\mathrm{TMTA}:\;\mathrm{TMT}-A & = & X_{\mathrm{the}\;\mathrm{presence}\;\mathrm{of}\;\mathrm{lacunes}\;}+X_{\mathrm{age}}+X_{\mathrm{sex}}+X_{\mathrm{BMI}}\\ & & +X_{\mathrm{hypertension}}+X_{\mathrm{disease}\;\mathrm{duration}}+X_{\mathrm{WHM}}\\ & & +X_{\mathrm{BG}-\mathrm{EPVS}}+a \end{array}$$

…

$$\begin{array}{ccc} \mathrm{Model}\;1\_\mathrm{MDS}-\mathrm{UPDRS}\mathrm{III} & = & X_{\mathrm{the}\;\mathrm{presence}\;\mathrm{of}\;\mathrm{lacunes}\;}+X_{\mathrm{age}}+X_{\mathrm{sex}}+X_{\mathrm{BMI}}\\ & & +X_{\mathrm{hypertension}}+X_{\mathrm{disease}\;\mathrm{duration}}+a \end{array}$$

$$\begin{array}{ccc} \mathrm{Model}\;2\_\mathrm{MDS}-\mathrm{UPDRS}\mathrm{III} & = & X_{\mathrm{the}\;\mathrm{presence}\;\mathrm{of}\;\mathrm{lacunes}\;}+X_{\mathrm{age}}+X_{\mathrm{sex}}+X_{\mathrm{BMI}}\\ & & +X_{\mathrm{hypertension}}+X_{\mathrm{disease}\;\mathrm{duration}}+X_{\mathrm{WHM}}\\ & & +X_{\mathrm{BG}-\mathrm{EPVS}}+a \end{array}$$

…

$$\begin{array}{ccc} \mathrm{Model}\;1\_\mathrm{MoCA} & = & X_{\mathrm{the}\;\mathrm{presence}\;\mathrm{of}\;\mathrm{lacunes}}+X_{\mathrm{age}}+X_{\mathrm{sex}}+X_{\mathrm{BMI}}\\ & & +X_{\mathrm{hypertension}}+X_{\mathrm{disease}\;\mathrm{duration}}+a \end{array}$$

$$\begin{array}{ccc} & & \mathrm{Model}\;2\_\mathrm{MoCA}=X_{\mathrm{the}\;\mathrm{presence}\;\mathrm{of}\;\mathrm{lacunes}\;}+X_{\mathrm{age}}+X_{\mathrm{sex}}+X_{\mathrm{BMI}}\\ & & +X_{\mathrm{hypertension}}+X_{\mathrm{disease}\;\mathrm{duration}}+X_{\mathrm{WHM}}+X_{\mathrm{BG}-\mathrm{EPVS}}+a \end{array}$$

…

And so on.

For dependent variables whose residuals did not conform to the normal distribution, data transformation was performed, including log transformation for TMT‐B and reciprocal transformations for TMT‐A, standing with eyes open, standing with eyes closed, tandem stance, and standing on one leg. The beta‐coefficients (β) and 95% confidence intervals (CI) of the independent factor *X*_the presence of lacunes_ for each of the 12 linear models of Model 1 and Model 2 were reported respectively in the table. Two‐tailed *p* values < .05 were considered statistically significant. The data with missing outcomes were removed from the analysis and the analysis with missing data imputation was also performed. All analyses were performed using SPSS Statistics version 25.0 software (IBM, Armonk, NY, USA).

## 3 RESULTS

### 3.1 Group comparisons of clinical characteristics, cognition and motor function

Ninety‐four patients were included in this study. As shown in Table 1, patients with lacunes showed higher body mass index and shorter disease duration than patients without lacunes (–1.70 [95% CI: –2.88 to –0.69] for body mass index and 16.5 [95% CI: 1.0 to 36.0] for disease duration). In addition, WMH and BG‐EPVS seemed to be more severe in patients with lacunes (*p* = .112 for WMH and *p* = .071 for BG‐EPVS). Other demographic profiles were comparable between groups.

Global cognitive function was comparable between groups (Table 2). However, compared with patients without lacunes, patients with lacunes spent more time on TMT‐A (–12.64 [95% CI: –22.50 to –3.00]). Regarding motor function, patients with lacunes showed slower 10MWT comfortable gait speed (0.08 [95% CI: 0.003 to 0.156]). Neither MDS‐UPDRS III nor postural control measurements differed between groups (Table 2). The results of missing data imputation with the mean were shown in Table A2.

**TABLE 2:** Comparison of cognition and motor function between groups

	PD without lacunes (n = 56)	PD with lacunes (n = 38)	p Value
MoCA ^b^	26 (5)	25 (4)	.237
TMT‐A, s ^b^ ^, #^	54.99 (31.64)	73.55 (32.11)	**.009**
TMT‐B, s ^b^ ^, §^	96.30 (59.50)	115.70 (103.05)	.138
SDMT ^a^ ^, *^	35 ± 9	32 ± 10	.108
MDS‐UPDRS III ^a^	28.4 ± 13.0	31.1 ± 8.2	.227
10MWT comfortable gait speed, m/s ^a^	1.25 ± 0.18	1.17 ± 0.18	**.042**
10MWT fast gait speed, m/s ^a^	1.67 ± 0.27	1.61 ± 0.25	.235
FTSTS, s ^b^	10.23 (3.11)	10.55 (3.59)	.190
Standing with eyes open, cm/s ^b^	2.64 (0.79)	2.5 (0.75)	.295
Standing with eyes closed, cm/s ^b^	2.80 (0.88)	2.66 (0.61)	.291
Tandem stance, cm/s ^b^	4.37 (1.78)	4.48 (2.62)	.419
Standing on one leg, cm/s ^b^	6.96 (3.60)	7.99 (4.37)	.165

^a^ Normally distributed data are expressed as the mean ± standard deviation.

^b^ Nonnormal data are reported as the median (interquartile range).

^#,§,*^There are two, one, and six missing data in the TMT‐A, TMT‐B, and SDMT, respectively. Bold values mean p < 0.05.

PD, Parkinson's disease; MoCA, Montreal Cognitive Assessment; TMT‐A, The Trail‐Making Test part A; TMT‐B, The Trail‐Making Test part B; SDMT, Symbol Digit Modalities Test; MDS–UPDRS III, Movement Disorder Society United Parkinson's Disease Rating Scale Part III; 10MWT, 10‐Meter Walk Test; FTSTS, five times sit‐to‐stand test.

### 3.2 Correlations between the number of lacunes and clinical assessments of PD

**FIGURE 2:**
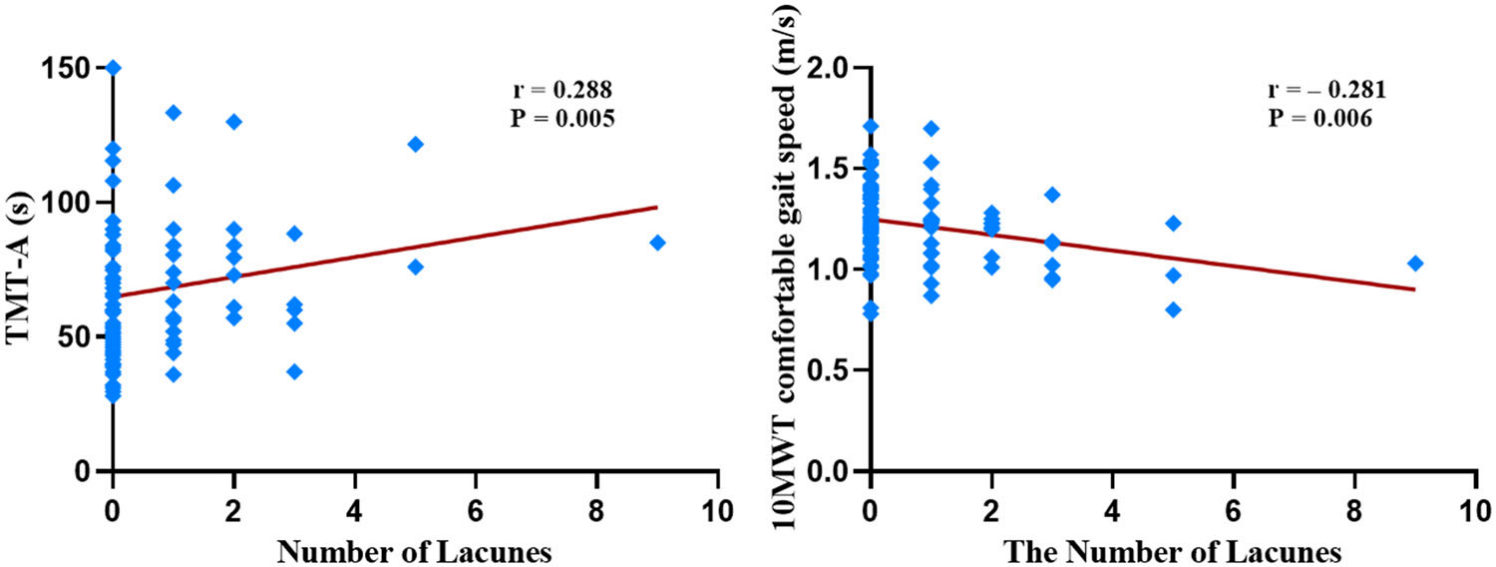
Correlations between the number of lacunes and clinical evaluations in patients with PD. PD, Parkinson's disease; TMT‐A, The Trail‐Making Test part A; 10MWT, 10‐Meter Walk Test

Spearman's correlation analysis showed that the number of lacunes were positively correlated with TMT‐A (*p* = .005, Figure 2). The number of lacunes were negatively related to 10MWT comfortable gait speed (*p* = .006, Figure 2). Correlation between the number of lacunes and postural control was not found in this study (Table 3). The results of missing data imputation with the mean were shown in Table A3.

**TABLE 3:** Correlations between the number of lacunes and clinical assessments in PD

	r	p Value
MoCA	–0.149	.151
TMT‐B, s	0.174	.095
SDMT	–0.208	.052
MDS‐UPDRS III	0.166	.111
10MWT fast gait speed, m/s	–0.124	.234
FTSTS, s	0.151	.146
Standing with eyes open, cm/s	–0.085	.413
Standing with eyes closed, cm/s	–0.104	.319
Tandem stance, cm/s	0.107	.304
Standing on one leg, cm/s	0.162	.118

PD, Parkinson's disease; r, correlation coefficient; MoCA, Montreal Cognitive Assessment; TMT‐B, The Trail‐Making Test part B; SDMT, Symbol Digit Modalities Test; MDS‐UPDRS III, Movement Disorder Society United Parkinson's Disease Rating Scale Part III; 10MWT, 10‐Meter Walk Test; FTSTS, five times sit‐to‐stand test.

### 3.3 Regression analysis of the presence of lacunes and clinical assessments of PD

The multivariate linear regression models showed that the presence of lacunes was associated with longer TMT‐A time after adjusting for age, sex, body mass index, and hypertension (–0.328 [95% CI: –0.007 to –0.001], Table 4). And the association was independent of the severity of the WMH and BG‐EPVS. We did not find any associations of the presence of lacunes, other cognition and motor symptoms after correcting for potential confounding factors (Table 4). The results of missing data imputation with the mean were shown in Table A4.

**TABLE 4:** Regression analysis of the presence of lacunes and clinical assessments in PD

Dependent variable	Model 1		Model 2	
	β (95% CI)	p Value	β (95% CI)	p Value
MoCA	–0.057 (–1.771, 1.076)	.629	–0.031 (–1.609, 1.232)	.792
TMT‐A, s ^#^	–0.328 (–0.007, –0.001)	**.003**	–0.342 (–0.007, –0.002)	**.003**
TMT‐B, s ^#^	0.116 (–0.048, 0.148)	.310	0.109 (–0.053, 0.147)	.351
SDMT	–0.090 (–5.816, 2.392)	.409	–0.084 (–5.681, 2.520)	.445
MDS‐UPDRS III	0.161 (–1.454, 8.851)	.157	0.149 (–1.758, 8.607)	.192
10MWT comfortable gait speed, m/s	–0.189 (–0.153, 0.010)	.084	–0.163(–0.144, 0.020)	.136
10MWT fast gait speed, m/s	–0.102 (–0.171, 0.063)	.360	–0.092 (–0.166, 0.069)	.415
FTSTS, s	0.112 (–0.584, 1.699)	.335	0.111 (–0.612, 1.720)	.347
Standing with eyes open, cm/s ^#^	–0.098 (–0.054, 0.020)	.373	–0.095 (–0.054, 0.022)	.395
Standing with eyes closed, cm/s ^#^	–0.045 (–0.042, 0.028)	.692	–0.057 (–0.045, 0.027)	.624
Tandem stance, cm/s ^#^	–0.142 (–0.054, 0.012)	.213	–0.138 (–0.054, 0.013)	.234
Standing on one leg, cm/s ^#^	–0.158 (–0.042, 0.005)	.129	–0.161 (–0.042, 0.005)	.128

^#^ Data that were transformed to make the residuals distribute normally. Bold values mean p < 0.05. Model 1 was adjusted for age, sex, body mass index, hypertension, and disease duration; Model 2 was further adjusted for white matter hyperintensity and enlarged perivascular space in the basal ganglia; β, standardized beta‐coefficients of the independent factor X _the presence of lacunes_ for each model after changing the dependent variables (Y) with those reported in the first column.

CI, confidence interval; MoCA, Montreal Cognitive Assessment; TMT‐A, The Trail‐Making Test part A; TMT‐B, The Trail‐Making Test part B; SDMT, Symbol Digit Modalities Test; MDS‐UPDRS III, Movement Disorder Society United Parkinson's Disease Rating Scale Part III; 10MWT, 10‐Meter Walk Test; FTSTS, five times sit‐to‐stand test.

## 4 DISCUSSION

To the best of our knowledge, this is the first study to investigate whether lacunes are independently associated with cognition and motor dysfunctions in PD. The main finding of the study was that the presence of lacunes was associated with poor attention, visual search and psychomotor speed in PD regardless of the severity of WMH and BG‐EPVS. We also found negative relationships of counts of lacunes, disease duration and gait speed, suggesting that lacunes may accelerate the progression of PD.

In patients with PD, H. M. Chen et al. (2021) reported that the number of lacunes was positively correlated with the severity of global cognitive impairment, while they did not conduct further analysis to control for confounding factors. Other studies failed to show a significant relationship of lacunes to global cognitive impairment after adjusting for confounders (Malek et al., 2016; Shibata et al., 2019), which was in line with our study. Some studies indicated that the influence of lacunes on cognitive function was restricted to specific domains. Lacunes were related to worse executive function and processing speed (Benjamin et al., 2014; Jokinen et al., 2011, 2020), but not for memory or global cognitive function (Jokinen et al., 2011, 2020). The TMT‐A is widely used as a measure of visual search, attention and psychomotor speed, and the TMT‐B additionally requires cognitive flexibility and set‐shifting abilities. A previous study conducted in China reported norms for TMT performance of individuals aged 66.2 ± 8.6 years, with norm values of 61.0 ± 26.5 for TMT‐A and 106.6 ± 57.6 for TMT‐B (Wei et al., 2018). Patients with lacunes in our study spent more time on the TMT‐A and TMT‐B than most Chinese elderly, which suggested that these patients might suffer from cognitive declines. After adjusting for potential confounders such as age, the severity of WMH and BG‐EPVS, we found that the presence of lacunes was still related to poor performance of TMT‐A in patients with PD, which has never been reported before. In nondisabled elderly subjects with leukoaraiosis, the presence of lacunes in the thalamus was associated with worse global cognitive function, executive function and psychomotor speed, independently of the extent of WMH (Benisty et al., 2009). Lacunes can independently predict executive functions over a 3‐year follow‐up, regardless of other imaging markers of cerebral small vessel disease (Jokinen et al., 2020). The mechanism behind the effect of lacunes on cognitive impairments remains unclear. Studies reported that thalamic and basal ganglia lacunes, particularly those in regions connecting to the prefrontal cortex, were associated with cognitive decline (Benjamin et al., 2014; Gold et al., 2005). What's more, lacunes correlated with hypometabolism in dorsolateral frontal cortex and metabolic activity in dorsolateral frontal cortex predicted cognitive decline (Reed et al., 2001; Reed et al., 2004). The above‐described studies indicated that lacunes may impair cognition via disrupting the corticostriatal‐thalamo‐cortical loops.

We found that patients with PD with lacunes showed shorter disease duration but slower gait speed than those without lacunes in our cohort, and we speculated that lacunes may worsen the symptoms of PD. H. Chen et al. (2020) found that the presence of lacunes was significantly associated with higher gait/posture subscores after adjusting for potential confounders. However, we failed to find an association between lacunes and postural instability/gait difficulty in patients with PD, which was accordance with previous studies (Malek et al., 2016; Y. Wan et al., 2019). Considering that clinical tests of balance and traditional measures of postural sway appear to be insensitive in identifying early changes of postural control in patients with PD (Kamieniarz et al., 2021), we used center of pressure displacement (a reliable measurement) (Terra et al., 2020) to quantify postural control in patients with PD. And we still did not find any relationships between lacunes and postural control in patients with PD. Cai et al. (2021) indicated that gait disorders in the elderly with cerebral small vessel disease is mediated by psychomotor speed and executive function. Lacunes may influence gait and balance via the following two mechanisms: (1) direct damage to motor pathways, such as the frontal lobe and basal ganglia; and (2) impairing the cognitive function (Su et al., 2022). In PD, impaired vision and cognition contribute independently to gait deficit (Stuart et al., 2016). Successful performance of the TMT‐A requires engagements of visual scanning, attention, and processing speed. Although we did not find direct relationships between lacunes and gait as well as postural control in PD, lacunes might indirectly contribute to gait and postural control dysfunctions via impairing visual scanning, attention, and processing speed functions. The relationship of lacunes, cognitive impairments, and gait disorders in PD deserves further investigation.

In this study, WMH and BG‐EPVS were considered as confounding factors between lacunes and cognitive as well as motor deterioration in PD. By controlling them, we could detect unique individual contributions of lacunes to cognitive and motor dysfunctions in PD. In addition, we quantitatively evaluated postural control rather than using gait difficulty/posture instability subscale to reduce subjective bias. However, many limitations of the present study should be considered. First, this is an exploratory observational study with a relatively small sample size. Further well‐designed studies with sufficiently large samples are needed. Second, we evaluated the imaging markers of cerebral small vessel disease manually, which is time consuming and susceptible to subjectivity. However, these semiquantitative methods are simple with good interrater reliability and easy to apply in clinical practice. Third, this study was cross‐sectional and did not address causal relationships. Fourth, some studies indicated that the effects of lacunes on cognition and motor function depend on their locations and number (Benisty et al., 2009; de Laat et al., 2010). We did not perform subgroup analysis to investigate the effects of lacunar location and count on cognitive and motor impairments in PD due to the small sample with lacunes, which should be considered in future studies. Finally, there may be other confounders that we didn't take into consideration, such as iron deposition (Ndayisaba et al., 2019), dyskinesia, and orthostatic hypotension (Pilotto et al., 2019).

In conclusion, lacunes were independently associated with worse visual scanning, attention, and processing speed in patients with PD. What's more, lacunes may accelerate the course of PD. Management of potential risk factors for lacunes and treatment of lacunes, together with early physical and cognitive training, may mitigate symptoms in patients with PD, which needs further study.

## CLINICAL TRIAL REGISTRATION

ChiCTR2000033768

## AUTHOR CONTRIBUTIONS

Author contributions included conception and study design (BF, JX, AL, ZJ, and KC), data collection or acquisition (ZJ, JF, LQ, CL, RW, YS, HY, and KC), statistical analysis (BF and KC), interpretation of results (BF and JX), drafting the manuscript work (KC), revising the manuscript critically for important intellectual content (BF, JX, and AL) and approval of final version to be published and agreement to be accountable for the integrity and accuracy of all aspects of the work (KC, ZJ, JF, LQ, CL, RW, YS, HY, AL, JX, and BF).

## CONFLICT OF INTEREST

The authors have no relevant financial or conflict of interest to disclose.

### FUNDING

This study was supported by the Science and Technology Development Fund of Beijing Rehabilitation Hospital, Capital Medical University (2019R‐006 for ZJ; 2020–069 and 2021‐011 for BF; 2020–051 for HY). The funding body had no role in protocol design, statistical analysis, and manuscript preparation.

### ETHICS STATEMENT

This study was approved by the Institutional Ethics Committee of Beijing Rehabilitation Hospital, Capital Medical University (approval No. 2020bkky010) and was performed in accordance with the Helsinki Declaration of 1975.

### DECLARATION OF PATIENT CONSENT

The authors certify that they have obtained all appropriate patient consent forms. In the form, the patients have given their consent for their images and other clinical information to be reported in the journal. The patients understand that their names and initials will not be published and due efforts will be made to conceal their identity.

### PEER REVIEW

The peer review history for this article is available at https://publons.com/publon/10.1002/brb3.2880

## Data Availability

The data that support the findings of this study are available from the corresponding author upon reasonable request

## 1

### 1.1

**TABLE A1:** Details of quantitative postural control assessment

Details of postural control assessment	
Task 1. standing with eyes open	Patients were asked to stand with their feet on the projected feet (**Supplementary Figure S1**) with eyes open for 30 s. Figure A1. The position of the projected feet on the force platform.
Task 2. standing with eyes closed	Patients were asked to stand with their feet on the projected feet with eyes closed for 30 s.
Task 3. tandem stance	Patients were required to place one foot in front of the other with eyes open for 10 s, with no distance between the hallux of the foot behind and the heel of the front foot.
Task 4. standing on one leg	Patients were instructed to stand with the preferred leg in the center of the platform with eyes open for 10 s.
Each task was repeated twice during the postural control assessment. The entire assessment was performed with a fixed sequence (standing with eyes open, standing with eyes closed, tandem stance, standing on one leg) and without any shoes on. Patients were instructed to look straight ahead with their head upright and their arms by their sides while standing as still as possible. To protect patients from falls or injury during the assessment, patients always wore a harness, and the evaluator paid close attention to patients throughout the assessment. The outcome measure was the displacement of the center of pressure (COP) (cm/s) during those four tasks. The lower the value, the better the postural control.	

**TABLE A2:** Comparison of cognitive function between groups

	PD without lacunes (n = 56)	PD with lacunes (n = 38)	p Value
TMT‐A, s ^b^	54.99 (31.64)	73.04 (31.57)	**.009**
TMT‐B, s ^b^	96.30 (59.75)	116.85 (100.02)	.121
SDMT ^a^	35 ± 8	32 ± 10	.107

^a^Normally distributed data are expressed as the mean ± standard deviation, and.

^b^Nonnormal data are reported as the median (interquartile range); TMT‐A has two missing data; TMT‐B has one missing data; SDMT has six missing data. Bold values mean p < 0.05.

TMT‐A, The Trail‐Making Test part A; TMT‐B, The Trail‐Making Test part B; SDMT, Symbol Digit Modalities Test.

**TABLE A3:** Correlations between the number of lacunes and cognitive function in PD

	r	p Value
TMT‐A, s	0.289	.005
TMT‐B, s	0.178	.086
SDMT	–0.199	.054

TMT‐A has two missing data; TMT‐B has one missing data; SDMT has six missing data.

PD, Parkinson's disease; r, correlation coefficient; TMT‐A, The Trail‐Making Test part A; TMT‐B, The Trail‐Making Test part B; SDMT, Symbol Digit Modalities Test.

**TABLE A4:** Linear regression for the associations between lacunes and clinical assessments in PD

Dependent variable	Model 1		Model 2	
	β (95% CI)	p Value	β (95% CI)	p Value
TMT‐A, s ^#^	–0.330 (–0.007, –0.001)	**.003**	–0.341 (–0.007, –0.002)	**.003**
TMT‐B, s ^#^	0.116 (–0.047, 0.147)	.312	0.109 (–0.052, 0.145)	.351
SDMT	–0.082 (–5.432, 2.410)	.446	–0.072 (–5.291, 2.649)	.510

^#^Data that were transformed to make the residuals distribute normally. Model 1 was adjusted for age, sex, body mass index, hypertension, and disease duration; Model 2 was further adjusted for white matter hyperintensity and enlarged perivascular space in the basal ganglia; Bold values mean p < 0.05. TMT‐A has two missing data; TMT‐B has one missing data; SDMT has six missing data; β, standardized beta‐coefficients of the independent factor X _the presence of lacunes_ for each model after changing the dependent variables (Y) with those reported in the first column.

CI, confidence interval; PD, Parkinson's disease; TMT‐A, The Trail‐Making Test part A; TMT‐B, The Trail‐Making Test part B; SDMT, Symbol Digit Modalities Test.
